# Supplementary figures and images for: Tunable theranostics: innovative strategies in combating oral cancer
Source: PeerJ. 2024 Jan 4;12:e16732. doi: 10.7717/peerj.16732 (PMC10771769; doi:10.7717/peerj.16732)

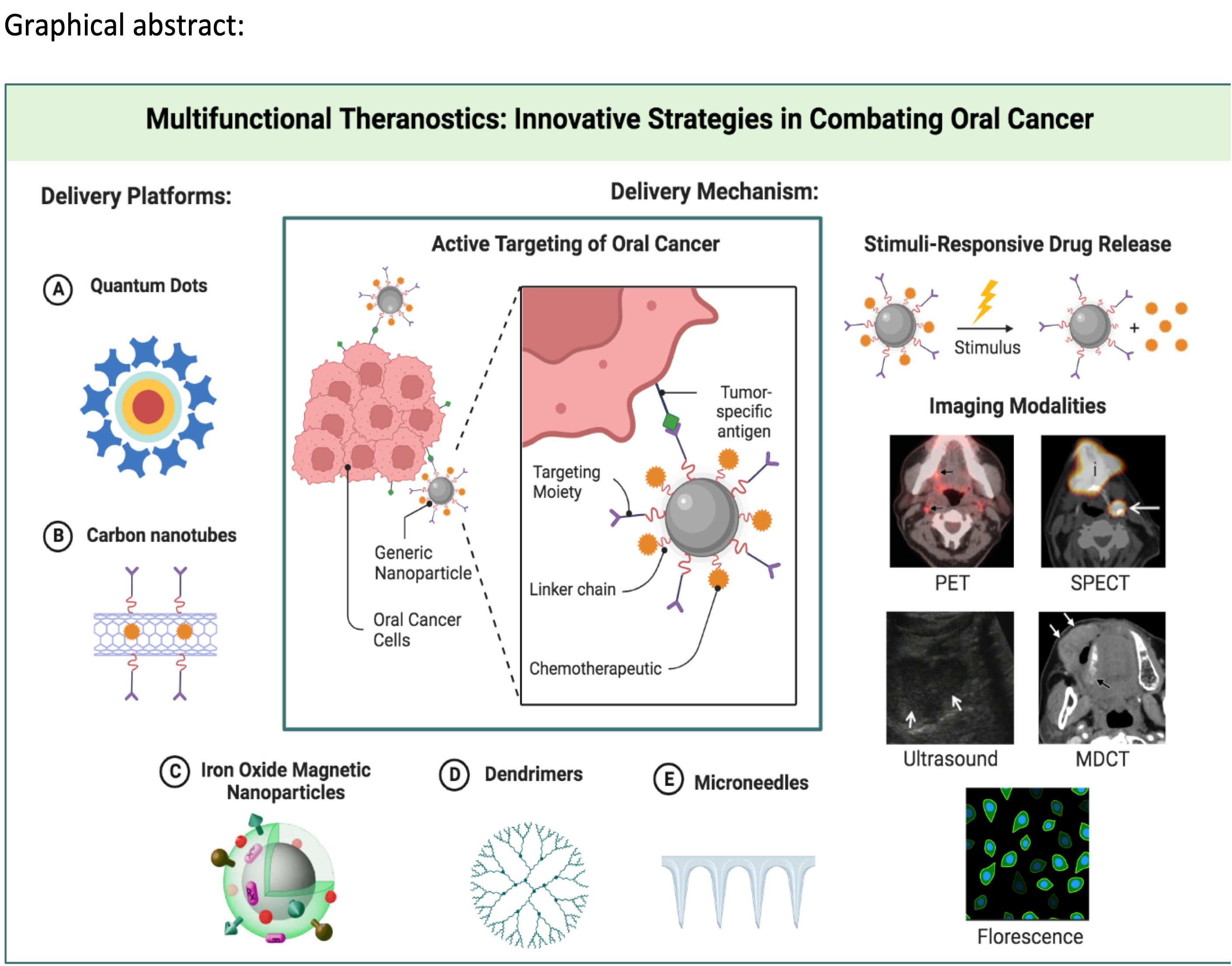

Supplement: Supplemental Information 1 [file peerj-12-16732-s001.png]
